# DeDoc2 Identifies and Characterizes the Hierarchy and Dynamics of Chromatin TAD‐Like Domains in the Single Cells

**DOI:** 10.1002/advs.202300366

**Published:** 2023-05-10

**Authors:** Angsheng Li, Guangjie Zeng, Haoyu Wang, Xiao Li, Zhihua Zhang

**Affiliations:** ^1^ State Key Laboratory of Software Development Environment School of Computer Science Beihang University Beijing 100191 P. R. China; ^2^ Zhongguancun Laboratory Beijing 100094 P. R. China; ^3^ CAS Key Laboratory of Genome Sciences and Information Beijing Institute of Genomics Chinese Academy of Sciences and China National Center for Bioinformation Beijing 100101 China; ^4^ School of Life Science University of Chinese Academy of Sciences Beijing 101408 P. R. China

**Keywords:** 3D genome, Hi‐C, hierarchy, single cell, TAD

## Abstract

Topologically associating domains (TADs) are functional chromatin units with hierarchical structure. However, the existence, prevalence, and dynamics of such hierarchy in single cells remain unexplored. Here, a new generation TAD‐like domain (TLD) detection algorithm, named deDoc2, to decode the hierarchy of TLDs in single cells, is reported. With dynamic programming, deDoc2 seeks genome partitions with global minimal structure entropy for both whole and local contact matrix. Notably, deDoc2 outperforms state‐of‐the‐art tools and is one of only two tools able to identify the hierarchy of TLDs in single cells. By applying deDoc2, it is showed that the hierarchy of TLDs in single cells is highly dynamic during cell cycle, as well as among human brain cortex cells, and that it is associated with cellular identity and functions. Thus, the results demonstrate the abundance of information potentially encoded by TLD hierarchy for functional regulation. The deDoc2 can be freely accessed at https://github.com/zengguangjie/deDoc2.

## Graphical abstract

DeDoc2 is a new generation TAD‐like domain (TLD) detection algorithm to decode the hierarchy of TLDs in single‐cell Hi‐C (scHi‐C) data via finding optimal structural entropy. TLDs detected by deDoc2 show that TLD hierarchy is associated with cellular identity and functions by regulating the expression of specific genes (case: astrocyte cell and APOE gene).

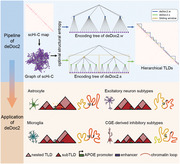

## 1 Introduction

The eukaryotic genome has a hierarchical configuration,^[^ ^1^, ^2^ ^]^ as revealed by imaging technologies^[^ ^3^ ^]^ and chromosome conformation capture‐based technologies,^[^ ^4^, ^5^, ^6^, ^7^, ^8^, ^9^, ^10^, ^11^, ^12^ ^]^ e.g., Hi‐C.^[^ ^8^ ^]^ These configurations, including chromosomal territories,^[^ ^8^, ^13^, ^14^ ^]^ A and B compartments,^[^ ^8^ ^]^ topologically associating domains (TADs),^[^ ^14^, ^15^ ^]^ compartment domains,^[^ ^16^ ^]^ or CTCF loop domains,^[^ ^17^ ^]^ and chromatin loops,^[^ ^17^, ^18^, ^19^ ^]^ have been routinely discussed.^[^ ^2^ ^]^ TADs may be one of the most investigated chromatin features in the literature. Studies have found that TADs are also organized in hierarchical fashion, e.g., the hierarchy of domains‐within‐domains (metaTAD) through TAD–TAD interactions at the large scale.^[^ ^20^, ^21^ ^]^ Hierarchical organization of TADs is functionally significant in that the hierarchical levels of metaTAD correlate with key epigenomic and expression features, and smaller subTADs are specifically associated with gene regulation.^[^ ^22^, ^23^, ^24^ ^]^ A long list of hierarchical TAD detection tools is available in the literature, such as Armatus,^[^ ^25^ ^]^ TADtree,^[^ ^26^ ^]^ GMAP,^[^ ^27^ ^]^ IC‐Finder,^[^ ^28^ ^]^ PSYCHIC,^[^ ^29^ ^]^ CaTCH,^[^ ^30^ ^]^ 3DNetMod,^[^ ^31^ ^]^ Matryoshka,^[^ ^32^ ^]^ deDoc,^[^ ^33^ ^]^ OnTAD,^[^ ^34^ ^]^ SpectralTAD,^[^ ^35^ ^]^ TADpole,^[^ ^36^ ^]^ HiCKey,^[^ ^37^ ^]^ and SuperTAD.^[^ ^38^ ^]^ However, all these tools were based on bulk Hi‐C data.

TAD‐like domains (TLDs) have been found in single cells.^[^ ^39^ ^]^ Tools for single‐cell TLD detection have been rapidly developed since the emergence of single‐cell Hi‐C (scHi‐C) technologies,^[^ ^40^ ^]^ including scHiCluster,^[^ ^41^ ^]^ deTOKI,^[^ ^42^ ^]^ Higashi,^[^ ^43^ ^]^ and HiCS.^[^ ^44^ ^]^ Some tools involved in data imputation were needed to solve the sparseness of single‐cell Hi‐C, e.g., scHiCluster,^[^ ^41^ ^]^ Higashi,^[^ ^43^ ^]^ and HiCS,^[^ ^44^ ^]^ while deTOKI^[^ ^42^ ^]^ utilized non‐negative matrix factorization (NMF) to address the problem. The advantage of NMF lies in its low rank representation, which retrieves key information embedded in the noisy sparse data. As a sparse non‐negative matrix, the sparsity of scHi‐C data can also be solved by NMF. However, all existing methods mainly utilized local information or arbitrarily borrowed information from neighboring cells, making it hard to explore higher‐level structures.

Given the large cell‐to‐cell variations of chromatin architecture observed in individual cells, whether exists of hierarchical TLDs or not remains a nontrivial question. In other words, the hierarchy of TADs observed in bulk Hi‐C could be a completely, or partially, emergent property of the cell population. That is, the dynamics of chromatin in single cells per se may generate, at least in part, the hierarchy.^[^ ^20^, ^21^ ^]^ Since the origin and dynamics of TADs are keys to understanding gene regulation,^[^ ^21^, ^45^ ^]^ it is essential to unravel the hierarchical nature of single‐cell TLD. However, a systematic survey of TLD hierarchy, including its dynamics, in single cells remains a major challenge in the field.

We recently developed deDoc,^[^ ^33^ ^]^ a tool which detects the hierarchical TAD structure with sparse Hi‐C data. The deDoc was based on the structural information theory,^[^ ^46^ ^]^ which measures the uncertainty embedded in the dynamics of a graph. Minimizing structural entropy is an intuitive way to decode the essential structure of a graph in which perturbations caused by random variation and noise have been reduced to a minimum. Thus, deDoc integrates the whole information in the graph, making the algorithm robust against data noise and sparseness. Recently, a tool named SuperTAD, which is based on structural entropy, also appeared in the literature. It is dedicated to the identification of hierarchical TADs using global minimization of structural entropy.^[^ ^38^ ^]^ However, neither deDoc nor SuperTAD work properly with single‐cell Hi‐C data.

Here, we present a new generation of TLD detector, deDoc2, which can reliably predict hierarchical TLD structures in single cells by dynamic programming. Compared to state‐of‐the‐art tools, deDoc2 not only outperforms its competitors in reliable TLD identification in single cells, but it is also robust to various data imputation algorithms. Using deDoc2, we found that hierarchical TLDs prevalent in single cells were dynamic, experiencing changes during cell cycle. We also found that TLD hierarchy was remarkably different among human brain prefrontal cortex cells. Here, we present examples, showing that both structure and hierarchy of TLDs may be subject to functional regulation in single cells. In short, deDoc2 opens the door to a systematic understanding of TLD hierarchy and, as such, it provides a new dimension by which to decipher the dynamics of single‐cell 3D chromatin structure.

## 2 Results

### 2.1 The deDoc2 Is a Single‐Cell Hierarchical TLD Predictor

**Figure 1:**
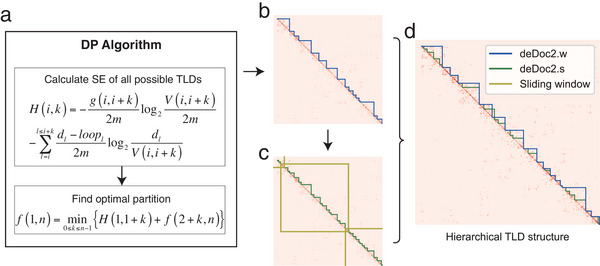
Flow chart of deDoc2. a) The dynamic programming algorithm used to minimize 2D structural entropy. b,c) An example of TLDs called by deDoc2.w and deDoc2.s, respectively, from the Hi‐C data in Rao et al. pertaining to cell 14 chromosome 18 within the range of 18–34 Mb. d) The hierarchical TLD structure comprises TLDs obtained from both deDoc2.w and deDoc2.s.

We developed a deDoc2 package to detect hierarchical TLDs with single‐cell Hi‐C data (**Figure** **1**). Similar to our previously developed deDoc,^[^ ^33^ ^]^ deDoc2 seeks to partition the genome into domains with minimal structural entropy. Briefly, the Hi‐C contact map was regarded as a network‐connected matrix, and the global optimal partition of the network with minimal 2D structural entropy was detected by a dynamic programming algorithm (Figure 1). However, deDoc2 goes beyond deDoc with its dynamic programming algorithm used to optimize 2D, but not high‐dimensional,^[^ ^38^ ^]^ structural entropy to achieve global optimization (Figure 1a). This allows a minimalist approach without losing key information.

The deDoc2 package consists of two predictors, deDoc2.w and deDoc2.s, to predict higher and lower level TLDs (Figure 1b,c). The deDoc2.w minimizes structural entropy of the Hi‐C contact map of whole chromosomes (Figure 1b), while deDoc2.s minimizes structural entropy in the matrices of sliding windows (10 Mb) along the genome (Figure 1c). Combining the results from the predictors, we can obtain the final two‐layer nested TLDs (Figure 1d). In addition to TLD prediction, the deDoc2 package provides a tool to determine proper binsize for the given Hi‐C data. Unlike deDoc which implements this feature with 1D structural entropy, deDoc2 uses normalized decoding information to optimize binsize (Figure S1, Supporting Information).

Next, we sought to assess the performance of deDoc2 by comparing it with eight state‐of‐the‐art algorithms, i.e., Insulation score (IS),^[^ ^47^ ^]^ deDoc,^[^ ^33^ ^]^ SpectralTAD,^[^ ^35^ ^]^ GRiNCH,^[^ ^48^ ^]^ deTOKI,^[^ ^42^ ^]^ scHiCluster,^[^ ^49^ ^]^ Higashi,^[^ ^43^ ^]^ and HiCS.^[^ ^44^ ^]^ These eight methods can be grouped into those mostly designed for sparseness (SpectralTAD and GRiNCH) or single cell (deTOKI, scHiCluster, Higashi, and HiCS). HiCS utilizes a scale parameter, *α*, to regulate the length of predicted TLDs. To enable a comprehensive evaluation, we tested the performance of HiCS using two values of *α*, namely, 1 and 0.3, which correspond to the default parameter (denoted as HiCS_de) and the parameter that produces TLDs of similar length to deDoc2.w (denoted as HiCS_sI), respectively. We first assessed them with downsampled ultrasparse Hi‐C data, followed by simulated and experimental single‐cell Hi‐C data. Sparsity was defined as the proportion of nonzero entries in the Hi‐C matrix. The assessment was made with and without data imputation. We mainly described the results from imputation‐free data, while similar performance with data imputation can be found in the Supporting Information (Figures S2 and S3, Supporting Information).

### 2.2 DeDoc2 Worked Well with Downsampled Bulk Hi‐C at the Single‐Cell Level

To assess the tools, we generated a series of downsampled data from Rao et al.,^[^ ^50^ ^]^ with sampling rates of 0.1%, 0.05%, and 0.025% and set default binsize = 40 kb. The dataset at the lowest sampling rate contains ≈0.23 m contacts, mimicking the sequencing depths of scHi‐C. For example, the median number of contacts in the data generated by Flyamer et al. was 0.339 m.^[^ ^51^ ^]^

**Figure 2:**
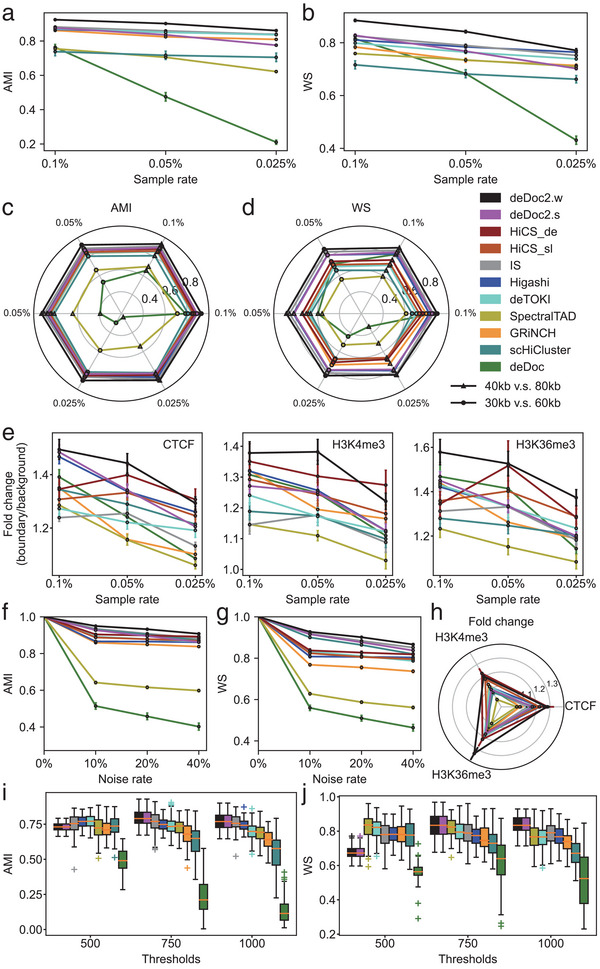
The assessment of performance of TLD callers, under various conditions, including downsampled, noisy, and simulated data based on the work of Rao et al. The sampling rates used were 0.1%, 0.05%, and 0.025%, while noisy data were generated by randomly introducing contacts into the 0.025% downsampled Hi‐C data, with ≈10%, 20%, and 40% more contacts added. Simulated data were generated using GM12878 data from Rao et al. within the chromosome region chr18:50–55 Mb. The results were reported as mean ± SEM, *n* = 23. a,b) Similarity indices, measured by the AMI and WS, respectively, between TLDs called from downsampled data. c,d) Robustness to binsize, indexed by AMI and WS, respectively, between TLDs called with different binsize (30 vs 60 kb and 40 vs 80 kb). e) Fold change of ChIP‐seq peak signals between TLD boundaries and regions away from boundaries. f,g) Similarity indices, measured by AMI and WS, respectively, between TLDs called from original and noisy Hi‐C data. h) Fold change of ChIP‐seq peak signals between TLD boundaries and regions away from boundaries with noisy data. i,j) Similarity indices, measured by AMI and WS, respectively, between TLDs called from simulated scHi‐C data at different simulation thresholds and bulk Hi‐C.

The deDoc2 outperformed the other tools in the following two respects. First, deDoc2 predicted TLDs more accurately than all other predictors. We took the TADs identified by full data with the predictors themselves as the gold standard and quantified the accuracy of predictions by the similarity, i.e., adjusted mutual information (AMI)^[^ ^52^ ^]^ and weighted similarity (WS).^[^ ^33^ ^]^ The predictions of deDoc2.w have the highest AMI and WS of all sample rates among all other algorithms, followed by Higashi, IS, deDoc2.s, and deTOKI (**Figure** **2**a,b). The order of the latter 4 algorithms was not consistent between the two accuracy indices; however, all are ranked in the top. HiCS is not included in this comparative analysis as it is incapable of identifying TADs using bulk Hi‐C data. Second, deDoc2 is more robust to binsize than the other algorithms. We compared the TLDs detected based on different binsizes, i.e., 30 versus 60 kb and 40 versus 80 kb. Again, we found that deDoc2.w had the highest AMI and WS among all algorithms for both binsize pairs, followed by IS, Higashi and deDoc2.s. The latter 3 are also ranked in the top (Figure 2c,d). Last, the characteristic binding of structural protein CTCF, or histone marks, was found to be more enriched in deDoc2‐predicted TLD boundaries than other tools. We compared the enrichment of ChIP‐seq peaks of H3K4me3, H3K36me3, and CTCF at the predicted TLD boundary regions (Figure 2e). The enrichment of all three marks is significantly higher in the boundaries predicted by deDoc2.w than in those predicted by the other tools at the sampling rates of 0.1% and 0.05%, and ranks at the top with HiCS_de at the sampling rate of 0.025% (Figure 2e). At the lowest sampling rate, Higashi, HiCS_sl, together with deDoc2.s have considerably high enrichment for those epigenetic marks. Taken together, our assessments suggest that deDoc2 can stably and accurately predict TLDs with ultralow resolution Hi‐C data.

### 2.3 DeDoc2 Is Robust Against Data Noise in Downsampled Hi‐C Data

Given the ultrasparse nature of scHi‐C data, TLD predictors may be sensitive to data noise. To assess the robustness of deDoc2 against data noise, we randomly modified 10%, 20%, and 40% of entries in the Hi‐C matrices with a replacement to mimic data noise in the downsampled data at the lowest sampling rate, 0.025% (see the Experimental Section). We found that deDoc2 is robust to noise because both AMI and WS of TLDs between noisy and original downsampled data decreased only slightly when noise rate increased (Figure 2f,g). Among all TLD predictors, deDoc2.w achieved the highest AMI and WS, followed by HiCS_de, deDoc2.s, and scHiCluster. The deDoc2.w and HiCS_de achieved enrichment much higher than all other predictors, and Higashi, scHiCluster, and deDoc2.s all achieved quite high enrichment at all noise rates (Figure 2h).

### 2.4 DeDoc2 Worked Well with Simulated scHi‐C Data

To assess the effect of cell‐to‐cell variance in the cell population to the performance of TLD predictors, we tested the predictors with simulated scHi‐C data. The simulation process was conducted on the basis of our previous work.^[^ ^42^ ^]^ To simplify the simulation, the structures in the ensemble were assumed to be evenly distributed in the cell population, and a 5 Mb‐long genome region, i.e., chr18:50–55 Mb, was randomly chosen as an example. As 5 Mb is smaller than the default sliding window size for deDoc2.s, the results for deDoc2.s and deDoc2.w were, in fact, identical, but we only reference deDoc2 in this section. Threshold (*D*) occurs in the simulation process (see the Experimental Section). We tested *D*s with 500, 750, and 1000, representing 20%, 40%, and 60% quantiles, respectively (Figure 2i,j). We simulated Hi‐C data from a 3D chromosome structure ensemble containing ≈100 single cells.^[^ ^42^ ^]^

The deDoc2 could accurately predict domain structures in simulated scHi‐C data. We generated simulated scHi‐C data ≈1000 and 0.35 m Hi‐C contacts for each single cells and the reference Hi‐C in that 5 m‐long chromosome region, respectively. We compared the accuracies (AMI and WS) of the predictions to the reference. For *D* = 750 and 1000, deDoc2 predicted the most accurate TLDs compared to the reference based on the highest AMI and WS (Figure 2i,j). For *D* = 500, deDoc2, together with other tools, except deDoc, predicted TLDs with almost indistinguishable AMI; however, the prediction of deDoc2 resulted in a substantially smaller WS compared to the other tools. This may have been caused by the bias to local contact of the Hi‐C matrix when *D* = 500. In this case, deDoc2 is prone to separate the large domains into smaller ones, leading to a larger number of predicted TLDs compared to the other tools (Figure S4a, Supporting Information). However, this issue can be resolved by data imputation (Figures S3b,c and S4b, Supporting Information), which is a common practice in single‐cell data analysis.^[^ ^21^ ^]^ In general, the performance of deDoc2 can be slightly improved by proper data imputation (see the Supporting Information). We were unable to evaluate HiCS as the current testing dataset contains fewer contacts than the minimum requirement for HiCS. Taken together, deDoc2 performed well in most cases in the simulated scHi‐C data.

### 2.5 DeDoc Predicts TLDs Well with Experimental scHi‐C Data

Next, we compared predictions among deDoc2, deDoc,^[^ ^33^ ^]^ SpectralTAD,^[^ ^35^ ^]^ GRiNCH,^[^ ^48^ ^]^ deTOKI,^[^ ^42^ ^]^ scHiCluster,^[^ ^49^ ^]^ Higashi,^[^ ^43^ ^]^ and HiCS,^[^ ^44^ ^]^ using three representative experimental scHi‐C datasets (hereinafter denoted as Tan's,^[^ ^53^ ^]^ Nagano's,^[^ ^54^ ^]^ and Lee's^[^ ^55^ ^]^) (Tables S1–S3, Supporting Information). We found the predictions of deDoc2, in most cases, to be among the top category.

**Figure 3:**
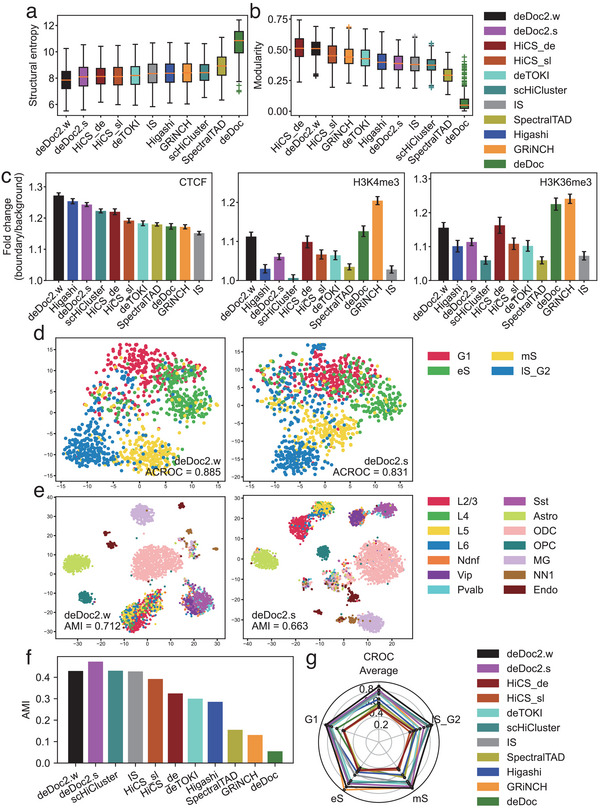
Assessment with experimental scHi‐C data. The datasets were Tan et al., Nagano et al., and Lee et al. a,b) The structural entropy and modularity of predicted TLDs in Tan's data. c) Fold change of ChIP‐seq peak signals between TLD boundaries and regions away from boundaries in Tan's data. The results were reported as mean ± SEM, *n* = 23. d,e) Cell embedding of TLDs predicted by deDoc2 with RWR imputation from PFC cells in Lee's data (4238 cells) and mES cells in Nagano's data (1137 cells), respectively. f) AMIs of embedding of TLDs from PFC cells in Lee's data (560 cells). g) CROCs of embedding of TLDs from mES cells in Nagano's data (400 cells).

As absent a gold standard for TLDs in single cells, we assessed performance indirectly. First, deDoc2 predicted TLDs with lower structural entropy and higher modularity (**Figure** **3**a,b). Structural entropy and modularity are two network properties that have been used to infer TADs for bulk Hi‐C data,^[^ ^31^, ^33^ ^]^ i.e., a better defined TAD will have smaller structural entropy^[^ ^33^ ^]^ and larger modularity.^[^ ^31^ ^]^ We tested the structural entropy and modularity of TLDs on 16 GM12878 cells from Tan's dataset and 20 randomly picked mouse ES cells from Nagano's dataset. Predictions of deDoc2.w and deDoc2.s had the first and second smallest structural entropies, respectively, in both datasets (Figure 3a; Figure S4c, Supporting Information). For modularity, the prediction of deDoc2.w and HiCS_de had the largest modularity in both datasets, while deDoc2.s‐predicted TLDs were similar to those of the other tools (Figure 3b; Figure S4d, Supporting Information). Second, CTCF and histone marks were enriched at the boundary regions of TLDs predicted by deDoc2, HiCS, Higashi and GRiNCH in experimental scHi‐C data (Figure 3c; Figure S4e, Supporting Information). For all marks in both datasets, none of the tools showed enrichment of predicted TLD boundaries. The prediction of deDoc2.w showed the highest enrichment among all three marks in at least one dataset. The prediction of HiCS_de showed the highest enrichment for H3K4me3 and H3K36me3 in Nagano's. Higashi's prediction showed the highest enrichment for CTCF in Nagano's. GRiNCH's prediction showed the highest enrichment for H3K4me3 and H3K36me3 in Tan's. Even in cases for which the predictions of deDoc2 did not show the highest enrichment, it could also be ranked at the top category by having enrichment comparable to the highest tool (Figure 3c; Figure S4e, Supporting Information).

Third, embedding scHi‐C data with deDoc2‐predicted TLDs results in satisfactory cell clustering (Figure 3d–g). We applied the eight algorithms on Nagano's and Lee's datasets and took TLD boundaries as text input. Then, we performed dimensionality reduction on their term‐frequency‐inverse document frequency values using TrunctedSVD.^[^ ^56^ ^]^ The last embedding could be visualized by t‐SNE (Figure 3d–g). In Lee's dataset, 4238 single cells are from the human prefrontal cortex (PFC) and were previously identified as having 14 cell types with CpG methylation levels.^[^ ^55^ ^]^ Consistent with clustering in the original work, the non‐neuronal brain cell types, i.e., Astro, ODC, OPC, and MG, can be clearly distinguished by both deDoc2.w and deDoc2.s (Figure 3d). In addition, clustering revealed two brain neuron subtype‐clusters, i.e., excitatory neuron subtypes, including L2/3, L4, L5, and L6, and inhibitory neuron subtypes, including Pvalb, Sst, Ndnf, and Vip. These two subtype‐clusters could not be clustered by chromatin contact alone in the original work.^[^ ^55^ ^]^ We used AMI to quantify the embedding. The deDoc2.s had the highest AMI value on Lee's data, followed by scHiCluster, deDoc2.w, and IS (Figure 3f). In Nagano's dataset, mouse embryonic stem (mES) single‐cell Hi‐C data come from four cell cycle stages,^[^ ^54^ ^],^ i.e., “G1” phase, “early‐S” phase, “mid‐S” phase, “late‐S/G2” phase, and “2n DNA” stages. As we failed to identify a sufficient number of TLDs from “2n DNA” stage, we excluded those data from subsequent analysis. After quality control, a total of 1171 cells were analyzed. The visualization showed a clear circular cell embedding with G1, eS, mS, and lS_G2 cells clustering in a clockwise manner for both deDoc2.w and deDoc2.s (Figure 3e). To compare this circular embedding with the other tools, we quantified the circularity with circular ROC (CROC).^[^ ^57^ ^]^ In 3 out of 4 stages, i.e., G1, mS, and lS_G2, and on average, embedding by deDoc2.w had the highest CROC value (Figure 3g). In eS, deDoc2.w came in second, along with GRiNCH, with comparable CROC. In G1 and mS, Higashi and GRiNCH came in second, along with deDoc.w, with comparable CROC, respectively. In general, the embedding by deDoc2, Higashi, GRiNCH, IS, and scHiCluster showed a circular property, suggesting that TLDs do, indeed, carry information for cell cycle. Taken together, our assessment suggests that deDoc2 works well with experimental scHi‐C data.

### 2.6 DeDoc2 Can Reveal Hierarchical TLD and Higher‐Level Structure in Single Cells

To assess the ability of deDoc2 to identify hierarchical chromatin structure at the single‐cell level, we quantified the hierarchy of the domain structure with three metrics, WS, and modularity^[^ ^58^ ^]^ and adjusted R^2^ (adjR^2^).^[^ ^34^ ^]^ The average modularity and adjR^2^ for all TLDs in a sample were denoted as TLD‐modularity and TLD‐adjR^2^, respectively. For a given list of TLDs, a better‐defined hierarchy will have larger TLD‐modularity and larger TLD‐adjR^2^.

With downsampled data at the sparse level of single‐cell Hi‐C, we found that deDoc2 identified hierarchical structure with larger TLD‐modularity and larger TLD‐adjR^2^ more stably when compared to three other hierarchy detectors, i.e., deDoc, SpectralTAD, and HiCS. SpectralTAD and HiCS are capable of identifying hierarchical structures at multiple levels. For the purposes of this study, we selected two levels that exhibited similar length distributions to the nested TLDs and subTLDs detected by deDoc2.

**Figure 4:**
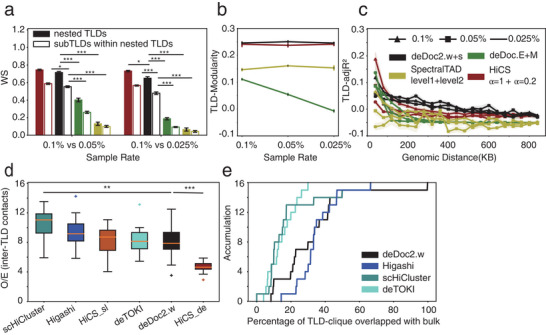
DeDoc2 reveals hierarchical TLD with ultrasparse Hi‐C data at single‐cell level. The results were reported as mean ± SEM, *n* = 23. a) The WS of nested TLDs and subTLDs within nested TLDs identified by different tools at various sampling rates. b) The TLD‐modularity of nested TLDs identified by different tools at various sampling rates. c) The TLD‐adjR^2^ of nested TLDs identified by different tools at various sampling rates. d) The enrichment of inter‐TLD contacts in TLD‐cliques based on TLD results of different tools in Tan's data. e) Percentage of TLD‐cliques overlapped with pooled single‐cell Hi‐C based on TLD results of different tools in Tan's data. Mann‐Whitney U test ***p < 0.001, **p < 0.01, *p < 0.05.

The deDoc2‐defined nested TLDs always yielded the top two WS and TLD‐modularity (**Figure** **4**a,b). For TLD‐adjR^2^, when the genomic distance is larger than 40 kb, deDoc2 was always substantially higher than deDoc, SpectralTAD, and HiCS (Figure 4c). Notably, when the genomic distance was smaller than 40 kb, deDoc or HiCS defined nested TLDs yielded the highest TLD‐adjR^2^. However, the length of TLDs and variation of adjR^2^s, as called by the two tools with genomic distance smaller than 40 kb, were also much shorter and larger than those called by deDoc2, respectively (Figure S5, Supporting Information). Given the sparseness at the downsampling rates we tested (0.1–0.025%), those small fragmented domains will be subject to severe stochastic fluctuation and thus unreliable. Therefore, deDoc2 can successfully define the hierarchy of a nested structure with ultrasparse Hi‐C data.

We evaluated the robustness of hierarchical TLD across single‐cell Hi‐C data in Tan's dataset. First, we examined the degree of cell‐to‐cell similarity of TLDs, subTLDs, nested TLDs, and subTLDs within nested TLDs and found that they were significantly higher than the shuffled control (the Experimental Section, Figure S6a–d, Supporting Information). Second, we observed that the level of similarity between cells and pooled or bulk datasets was higher than that between cells (Figure S6a–d, Supporting Information). Moreover, the degree of similarity in the TLD hierarchy (Figure S6c,d, Supporting Information) was lower than that in the TLD structure (Figure S6a,b, Supporting Information) across the single cells, which is biologically reasonable. Collectively, these findings suggest that hierarchical TLDs may relatively robust exist in single cells.

Dedoc2 can also identify higher‐level chromatin structure of TLDs, e.g., TLD‐cliques. TAD‐cliques comprise a higher level of chromatin structure than nested TADs found in bulk Hi‐C data.^[^ ^59^ ^]^ So, we asked if such higher‐level chromatin structure exists in single cells. Given the capacity of detecting TLDs in single cells, we expect well‐defined single‐cell TLD‐cliques to meet the following requirements. First, they should have a clean TLD pattern, i.e., as few inter‐TLD interactions as possible, given the significantly enriched inter‐TLD interactions among TLDs in the clique. Second, they should be very accurate, considering consistency with bulk TAD‐cliques in the cell population, cumulatively. We composed a pipeline to identify TLD‐cliques at the single‐cell level with predefined TLDs as input (the Experimental Section). We compared the cliques identified using TLDs called by deDoc2.w, scHiCluster, deTOKI, Higashi, and HiCS with Tan's dataset.^[^ ^53^ ^]^ Within the cliques identified, HiCS_de showed the lowest O/E inter‐TLD interactions (the Experimental Section), followed by deDoc2.w, deTOKI, HiCS_sl, and Higashi (Figure 4d). Thus, HiCS_de and deDoc2.w‐defined TLD‐cliques are significantly cleaner than those of the other three tools in the single cells we examined. However, HiCS_sl and HiCS_de exhibited notable differences in performance, indicating that the efficacy of HiCS is highly sensitive to the selection of parameters. We further verified the accuracy of the identified TLD‐cliques by overlapping them with bulk TLD‐cliques called from the pooled single‐cell Hi‐C data. Altogether, deDoc2.w and Higashi identified the highest proportion of bulk TLD‐cliques, higher than deTOKI and scHiCluster by more than 50% (Figure 4e). HiCS was not included in this comparison as it is incapable of identifying TADs using bulk Hi‐C data. These results show that deDoc2 can identify nested and higher‐level structures in the hierarchy of chromatin architecture at the single‐ cell level.

### 2.7 Hierarchical TLD Structure Is Highly Dynamic during Cell Cycle and among Human Brain Prefrontal Cortex Cells

**Figure 5:**
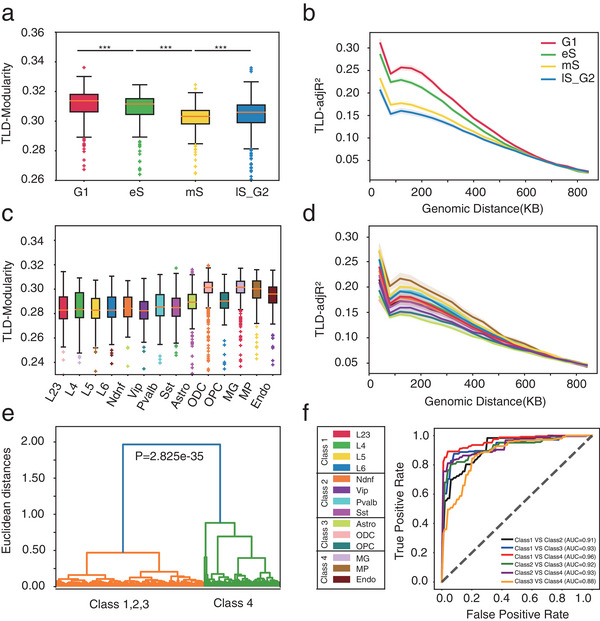
TLD structure is highly dynamic during cell cycle and among human brain prefrontal cortex cells. a,b) The TLD‐modularity and TLD‐adjR^2^ values, respectively, were predicted by deDoc2 using RWR imputation in Nagano's data of mouse embryonic stem (mES) cells, consisting of 1137 cells. Mann‐Whitney U tests revealed significant differences between groups (***p < 0.001, **p < 0.01, *p < 0.05). The solid line and shadow in b represent the mean ± SEM. c,d) The TLD‐modularity and TLD‐adjR^2^ values, respectively, were predicted by deDoc2 using RWR imputation in Lee's data of prefrontal cortex (PFC) cells, comprising 4238 cells. The solid line and shadow in (d) represent the mean ± SEM. e) Hierarchical classification of PFC cells in Lee's data was performed, with 30 cells randomly selected from each cell type, resulting in a total of 420 cells (hypergeometric tests). f) SVM classification of PFC cells in Lee's data was conducted, with 200 cells randomly selected from each cell type class, resulting in a total of 800 cells.

To investigate the dynamic hierarchical structure of chromosome architecture in single cells, we applied deDoc2 to two public single‐cell Hi‐C datasets, i.e., the cell cycle^[^ ^54^ ^]^ and human brain prefrontal cortex cells.^[^ ^55^ ^]^ We found that the nested TLDs dramatically changed among cell types in both datasets. From G1 into early and middle S stage, both modularity and TLD‐adjR^2^ gradually decreased (**Figure** **5**a,b), while from mS to lS_G2, they increased and continued to drop for modularity and TLD‐adjR^2^, respectively. Nevertheless, the absolute difference of TLD‐adjR^2]^ between mS and lS_G2 (0.145) was much weaker than the changes among earlier stages, i.e., 0.380 and 0.295 between G1 and both early S and middle S, respectively (Figure 5b). Interestingly, we found that nested TLDs not only varied substantially among cell types in the human brain prefrontal cortex (Figure 5c,d), but they might also be used to classify cell types. Using hierarchical clustering, we found that single cells could be roughly classified into neural and non‐neural categories by their modularity and TLD‐adjR^2^ (Figure 5e). Moreover, the two features could also largely pairwise distinguish the four categories of cells found in the prefrontal cortex (glutamatergic, GABAergic, non‐neuronal, and non‐neural^[^ ^60^ ^]^).

**Figure 6:**
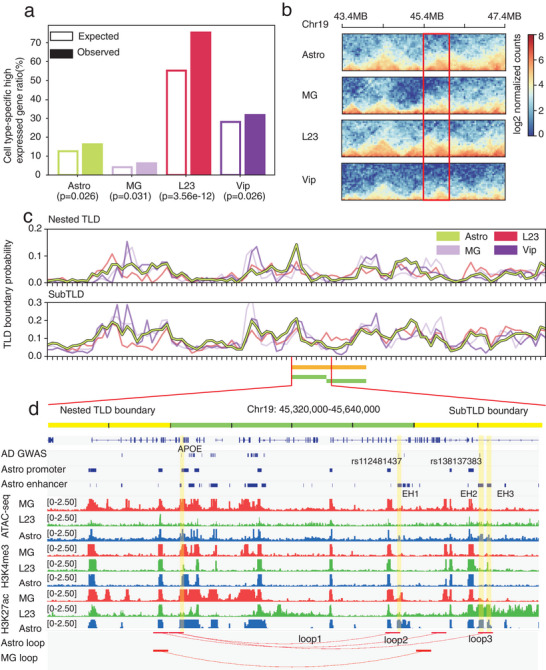
TLD structure related to cellular functionality. a) Cell type‐specific highly expressed gene distribution of cell type‐specific boundary (observed) and all highly expressed genes (expected) (hypergeometric test). b) Pooled single‐cell Hi‐C matrix of four cell types at chr19:43400000‐47400000, the red frame refers to the Astro specific nested TLD structure. c) Nested TLD and subTLD boundary probability at chr19:43400000‐47400000. d) Epigenetic data and loop identified by SnapHi‐C around the APOE gene and associated enhancers.

Thus, our analysis may imply that the hierarchical structure of TLD nesting may carry key information for cell identity with concomitant functional implications. For example, cell type‐specific genes were associated with cell type‐specific nested TLD boundaries (**Figure** **6**a). We picked one representative cell type from each of the four cell classes, i.e., L2/3, Vip, Astro, and MG, and defined their cell type‐specific highly expressed genes and nested TLD boundaries (the Experimental Section). We found that cell type‐specific highly expressed genes were significantly enriched in cell type‐specific nested TLD boundaries (Figure 6a). To illustrate the functional implication for the hierarchical TLD, we cited apolipoprotein E (APOE). APOE is a key gene found to be associated with Alzheimer's disease (AD).^[^ ^61^ ^]^ The APOE gene is highly expressed in Astro cells (Figure S7, Supporting Information) and locates in an Astro cell‐specific nested TLD boundary. Two Astro cell‐specific loops (loop1, loop3) in the locus link the APOE promoter to three active enhancers (EH1, EH2, EH3) identified by SnapHiC.^[^ ^62^ ^]^ It is known that Astro cells are associated with AD.^[^ ^63^, ^64^, ^65^ ^]^ EH1, EH2, and EH3 are also located around the Astro‐specific subTLD boundary, EH1 and EH2 carry AD‐associated GWAS SNPs (rs112481437, rs138137383) (Figure 6b–d). Thus, Astro‐specific EH1, EH2–APOE interaction between hierarchical TLD boundaries associated with the SNP toward AD is plausible. Needless to say, further detailed study is required to corroborate this speculation. Taken together, the hierarchical structure of chromosome architecture is a characteristic feature for single cells and may have profound effect on their cellular functionality.

## 3 Discussion

In this work, we present a novel algorithm, deDoc2, to detect TLDs at single‐cell level. Compared to its predecessor, deDoc, which reached a local optimal of structure entropy, we implemented a dynamic programming algorithm to approach the global optimal of structure entropy in deDoc2. A recently published TAD caller, SuperTAD, also employed dynamic programming to approach the global optimal of structural entropy. The deDoc2 and SuperTAD differ in that they minimize 2D and high‐dimensional structural entropy, respectively. SuperTAD requires several orders of magnitude higher CPU time and memory space to solve high‐dimensional structural entropy (Table S4, Supporting Information). Moreover, high‐dimensional entropy calculated from ultrasparse scHi‐C data may be less reliable. Therefore, SuperTAD may not be suitable for scHi‐C data analysis. Compared to state‐of‐the‐art tools, deDoc2 exhibits first‐class performance on the metrics we assessed in the downsampled, simulated, and experimental scHi‐C data.

The deDoc2 distinguished itself most from peer predictors with several unique features. First, deDoc2 decodes TLD from single‐cell Hi‐C data with no need of data imputation, while most state‐of‐the‐art TLD predictors do.^[^ ^41^ ^]^ In the ideal scenario, the imputation process does not introduce new information to the matrix regarding chromatin structure. However, the ultrasparseness of scHi‐C data made it plausible that certain artifacts may emerge from stochastic fluctuation. Thus, a method that does not involve data imputation may be less subject to the risk of false positives. Because the definition of structure entropy fully utilized the data in the whole contact matrix, deDoc2 achieved similar, even advanced, performance over imputation‐based TLD predictors (Figures S2 and S3, Supporting Information).

Second, deDoc2 can reveal the structural hierarchy of TLDs. The hierarchical structure of TADs is a common property seen in bulk Hi‐C data. However, to the best of our knowledge, it has been rarely discussed in the literature for single‐cell Hi‐C data, and the question may not be trivial. For example, whether the hierarchy seen in bulk Hi‐C is an emerging property of heterogeneous cell population, or it does, indeed, exist in individual cells, remains largely unexplored. Our method showed a clear hierarchy in single‐cell Hi‐C data, and the brief examples we showed in the present work suggested that this hierarchy in single cells may carry fundamental biological functions. Thus, to further reveal the mechanisms of higher‐level 3D genome folding, the dynamics and functions of hierarchical domain structure at the single‐cell level will be essential. The experiments we showed here suggested that deDoc2 is a unique predictor in the spectrum of single‐cell Hi‐C data analytic tools.

The TLD identified by deDoc2, as well as by other tools, exhibits remarkable heterogeneity, reflecting the highly dynamic nature of chromatin structure at the TLD level and beyond. Several potential mechanisms have been identified in the literature, such as the transition between lamina‐associated domains and nucleolus‐associated domains,^[^ ^66^ ^]^ and the dynamic behavior of chromatin regulators, including polycomb‐bodies^[^ ^67^ ^]^ and loop‐forming extrusion of chromatin fiber.^[^ ^18^ ^]^ Deciphering the mechanisms behind cell‐to‐cell variations in TLD is an intriguing avenue for further research. For instance, the monosided loop extrusion model may result in a relatively stable CTCF binding TLD boundary, juxtaposed to another more variable boundary. A systematic survey followed by experimental validation could be a promising direction for future investigations.

The integration of single‐cell multiomics data is a vital area of research, yet many challenges remain. First, as previously discussed, chromatin architecture is highly dynamic and exhibits considerable variability between cells. Similarly, other single‐cell features, such as chromatin accessibility (scATAC), chromatin chemical modification status (scChIP), and transcription (scRNA), are also highly dynamic and may not be entirely synchronized. Therefore, single‐cell multi‐omics technologies are critical to generating reliable reference datasets for any data integration methods. Second, sophisticated algorithms are required to deal with sparse, noisy, and heterogeneous multimodal single‐cell data. Recent progress in deep learning‐based AI technologies has proven to be impressively powerful in deciphering such multimodal big data.^[^ ^68^ ^]^ However, these methods remain weak in terms of interpretability. On the other hand, deDoc2 as an example, the methods that have information theory base not only performed well, and has much higher interpretability.

## 4 Conclusions

We present a newly developed single‐cell Hi‐C data analysis tool termed as deDoc2. Developed from structural information theory, deDoc2 not only outperformed state‐of‐the‐art tools for TLD detection, but it also has the unique feature of detecting the hierarchy of chromosome domain structure in single cells without data imputation. Finally, we demonstrated that higher‐level single‐cell 3D chromatin structure dramatically changes during cell cycle and among cell types and that this dynamism is tightly associated with gene expression changes, implying its functionality.

## 5 Experimental Section

### Structural Information Theory

Structural information theory^[^ ^46^ ^]^ measures and decodes information embedded in a system consisting of many bodies together with interactions among the individuals of the several bodies.

Hi‐C data can be interpreted as a weighted graph, and the TLD detection problem can be viewed as a graph partitioning problem. As in deDoc, TLDs are sought to detect by structural information theory.

A fundamental concept in structural information theory is the notion of encoding a tree, i.e., a connected graph with no cycles. In fact, encoding a tree is both mathematical model and data structure of the hierarchical abstracting of a graph.

### Encoding Tree

Structural information theory measures the information embedded in a graph that is decoded by an encoding tree. An encoding tree *T* of a graph *G*(*V*, *E*) is defined as follows.

1) For every tree node *α* ∈ *T*, a vertices set *T*_*α*_ ∈ *V* is associated with it, and the immediate successors of *α* are labeled by *α*^ ⟨0⟩, *α*^ ⟨1⟩, ··· *α*^ ⟨*k*⟩. It is said that *α* is the codeword of the set *T*_*α*_, and that *T*_*α*_ is the marker of *α*.
2) The root of *T* is an empty string *λ*, associated with the vertices set *V*, written as *T*_*α*_ = *V*.
3) For every *α* ∈ *T*, if *β*_0_, *β*_1_,⋅⋅⋅, *β*_*k*_ are all immediate successors of *α* in *T*, then $\left\{T_{\beta_{0}},T_{\beta_{1}},\cdot \cdot \cdot T_{\beta_{k}}\;\right\}$ is a partitioning of *T*_*α*_.
4) For every leaf node *γ* ∈ *T*, *T*_*γ*_ contains only one vertex of *V*.

The structural entropy of graph *G* given by an encoding tree *T* is defined as

(1) $$H^{T}\left(G\right)=\sum_{\alpha \in T,\;\alpha \neq \lambda }-\frac{g_{\alpha }}{2m}\log_{2}\frac{V_{\alpha }}{V_{\alpha^{-}}}$$

where *α* is a tree node of the encoding tree *T*, *λ* is the root of *T*, *α* ^−^ is the immediate predecessor of *α*, *g*_*α*_ is the cut of *α*, i.e., the number of edges between the vertices in and not in vertices *T*_*α*_, *V*_*α*_ is the volume of *α*, i.e., the sum of degrees of all vertices in *T*_*α*_, and *m* is the sum of the edges, sum(*E*).

The structural entropy of *G* is min_*T*_{*H^T^* (*G*)}, and the *k*‐dimensional structural entropy of *G* is min_*T*_{*H^T^* (*G*)}, where *T* has a height restriction of at most *k*.

### Decoding Information

Given a graph *G* and an encoding tree *T* of *G*,

i) the metric *H* ^1^(*G*), i.e., the 1D structural entropy of *G*, is the total amount of uncertainty that is embedded in *G* and
ii) the metric *H^T^* (*G*) is the amount of uncertainty that is embedded in the system obtained from *G* by the hierarchical abstracting given by *T*.

Therefore, the information decoded by the hierarchical abstracting *T* of encoding tree *T*, referred to as the decoding information of encoding tree *T* from *G*, is

(2) $$D^{T}\left(G\right)=H^{1}\left(G\right)-H^{T}\left(G\right)$$

The decoding information of *G* is max_*T*_{*D^T^* (*G*)}, and the *k*‐dimensional decoding information of *G* is max_*T*_{*D^T^* (*G*)}, where *T* has a height restriction of at most *k*. *D^T^* (*G*) is the metric of the information that is decoded by the strategy of hierarchical abstracting given by encoding tree *T* from graph *G*.

### DeDoc2

Aiming to seek the optimal partition of the genome, deDoc2 employed a dynamic programming algorithm to minimize 2D structural entropy. The deDoc2 package has three components. DeDoc2.w and deDoc2.s predict the global and local partition of the genome, respectively, and deDoc2.binsize determines the optimal binsize with normalized decoding information.

### DeDoc2.w: Minimize 2D Structural Entropy by Dynamic Programming Algorithm

Given a graph *G*, an encoding tree *T* with height 2 of *G* is equivalent to a vertex partitioning of *G*.

DeDoc2.w predicts the optimal partition *P* = {*X*_1_,*X*_2_,⋅⋅⋅, *X_L_*}, where each *X_i_* is a collection of continuous bins representing a TLD in the genome with minimal 2D structural entropy. By the definition of structural entropy of a graph under an encoding tree, the 2D entropy of *G* given by *P* can be expressed as

(3) $$H_{p}\left(G\right)=\sum_{j=1}^{L}\left(-\frac{g_{j}}{2m}\log_{2}\frac{V_{j}}{2m}-\sum_{i\in X_{j}}\frac{d_{i}-{\mathrm{loop}}_{i}}{2m}\log_{2}\frac{d_{i}}{V_{j}}\right)$$

where *d_i_* is the degree of node *i*, loop_*i*_ is the weight of self‐loop edges of node *i*, i.e., the *i*th value of diagonal, *g_j_* is the cut of TLD *j*, *V_j_* is the volume of TLD *j*, and *L* is the number of partitions. When predicting TLDs of a chromosome, if *k* + 1 bins is put from bin *i* to bin *i* + *k* (bin *i* and bin *i* + *k* included) in a TLD, the 2D structural entropy of this TLD is defined as

(4) $$H\left(i,k\right)=-\frac{g\left(i,i+k\right)}{2m}\log_{2}\frac{V\left(i,i+k\right)}{2m}-\sum_{l=i}^{l\leq i+k}\frac{d_{l}-{\mathrm{loop}}_{l}}{2m}\log_{2}\frac{d_{l}}{V\left(i,i+k\right)}$$

where *g*(*i*, *i* + *k*) is the cut of this TLD, and *V*(*i*, *i* + *k*) is its volume. The recurrence relation can be written as

(5) $$f\left(1,n\right)=\min_{0\leq k\leq n-1}\left\{H\left(1,1+k\right)+f\left(2+k,n\right)\right\}$$

where *f*(1, *n*) is the 2D structural entropy of a graph that includes nodes from 1 to *n*. To speed up the algorithm, the length of TLDs was limited within 10 Mb, i.e., $0\leq k\leq \min(\frac{10\;\mathrm{Mb}}{\mathrm{binsize}},\;n-1)$, in the recurrence relation equation above.

There are *n* possible values for *k* to choose and *n* possible *H*(1, 1 + *k*) terms, and *H*(1, 1 + *k*) can be calculated in *O*(1) time, so the time complexity of deDoc2.w is written as *O*(*n* ^2^). With the limitation of TLD length within 10 Mb, the possible value of *k* becomes $\frac{10\;\mathrm{Mb}}{\mathrm{binsize}}$, and the time complexity becomes $O(\frac{10\;\mathrm{Mb}}{\mathrm{binsize}}n)$, which is linear to the length of the chromosome.

To accommodate isolated vertices, which are usually seen in sparse Hi‐C data, a self‐loop value $r=\frac{2m}{n_{0}}$ is added, where *n*_0_ is the number of nonisolated vertices in the matrix, to increase the aversion of these nodes to form communities (see the Supporting Information).

### DeDoc2.s: Minimize 2D Structural Entropy in Sliding Windows by Dynamic Programming Algorithm

When 2D structural entropy minimization is applied to a smaller matrix, the TLD structure will be smaller. A sliding window is used to split the Hi‐C contact matrix and deDoc2.w is applied to the split matrix to get smaller TLDs and a hierarchical structure with TLDs is formed, which, in turn, is predicted by deDoc2.w. The default window size is set to be 10 Mb, which means $\frac{10\;\mathrm{Mb}}{\mathrm{binsize}}$ bins in the matrix. The first sliding window starts from the first bin and ends at $\frac{10\;\mathrm{Mb}}{\mathrm{binsize}}$ bin. After TLDs in the first window are predicted by deDoc2.s, the window slides to the first bin of the last TLD are predicted to keep potential TLDs from being cut by the sliding window. Repeating this to the end of the contact matrix, the predicted TLDs are obtained.

The time used for predicting TLDs in one sliding window is $O({\left(\frac{10\;\mathrm{Mb}}{\mathrm{binsize}}\right)}^{2})$, where $\frac{10\;\mathrm{Mb}}{\mathrm{binsize}}$ is the number of nodes in a sliding window, and the time complexity of deDoc2.s is written as $O(k{\left(\frac{10\;\mathrm{Mb}}{\mathrm{binsize}}\right)}^{2})$, where *k* is the number of sliding windows.

### DeDoc2.Binsize: Normalized Decoding Information for Binsize Determination

Decoding information of a graph measures the maximum amount of information that can be eliminated by an encoding tree such that a graph with larger decoding information implies a better community structure. In particular, in the case of Hi‐C data, larger decoding information implies a better TLD structure. The aim is to choose a proper binsize with which to build a graph that best shows the TLD structure in Hi‐C data. 2D structural entropy was only calculated in this paper, so *D* ^2^(*G*) was chosen as the decoding information for binsize determination. For simplicity, the encoding tree obtained by deDoc2.w was used to calculate the decoding information since deDoc2.w can obtain the optimal 2D encoding tree that most minimizes the structural entropy of the graph. Because decoding information is not comparable between binsizes, a way was searched to normalize the decoding information.^[^ ^69^ ^]^ To do this, the residual entropy, i.e., the decoding information of a partition, was normalized by dividing the 1D structural entropy of the graph. Here the same normalization was done and the normalized decoding information (NDI) was defined as

(6) $${\mathrm{NDI}}^{2}\left(G\right)=\frac{H^{1}\left(G\right)-H^{2}\left(G\right)}{H^{1}\left(G\right)}$$

A binsize is proposed to choose which maximizes the NDI of the Hi‐C contact matrix. A binsize is chosen so that its associated Hi‐C graph has the minimum normalized SE (nSE) among all stable binsizes.^[^ ^33^ ^]^ Comparing NDI and nSE, it is found that if nSE is minimum, the NDI is maximum among binsizes, so NDI and nSE are consistent with each other. However, it is worth noting that 2D structural entropy is calculated by deDoc(E) in nSE, while it is calculated by deDoc2.w in NDI; thus, the resulting structural entropy can be slightly different.

### Random Walk with Restart (RWR) Imputation

RWR provides a good relevance score between two nodes in a weighted graph, and it has been used in numerous settings.^[^ ^70^ ^]^

For a sparse Hi‐C matrix *M* to be imputed, it is normalized into a matrix *C* by sqrtVC normalization,^[^ ^50^ ^]^ which ensures that the resulting RWR imputation can converge and keep *C* symmetric. The imputed matrix *Q_t_* is calculated recursively as

(7) $$Q_{t}=\left(1-\alpha \right)\;Q_{t-1}C+\alpha I$$

where *Q*_0_ = *I*, and *α* is the restart probability. The random walk stops when ∥*Q_t_* − *Q*_*t* − 1_∥_2_ ≤ 10^−2^, which means that the process has converged.

### Adding Arbitrary Noise

Noise was introduced to the Hi‐C matrix as follows.^[^ ^71^ ^]^ For a given Hi‐C matrix (*N × N*), a proportion (*P_n_*) of entries were randomly selected with replacement and a constant of one added. As the selection was with replacement, the numbers added into the matrix follow binomial distribution *B* (*n*, *p*), where *n* is the number of entries to select and *p* is the 1/*N* ^2^. The *P_n_* was set to be 10%, 20%, and 40% of the number of contacts in the original downsampled Hi‐C matrices (0.025%) to form three noise rates. Considering that the contact probability of Hi‐C matrices decreases dramatically as the genomic distance increases, the contact probability of each secondary diagonal was calculated using the method in Boost‐HiC^[^ ^72^ ^]^ and entries with the replacement of each secondary diagonal were randomly selected separately according to its contact probability. To avoid adding noise to unmappable regions, the rows/columns with at least one nonzero entry were only considered in the downsampled Hi‐C matrices.

### The Simulation of scHi‐C Data

scHi‐C data were simulated according to the previous work.^[^ ^42^ ^]^ Briefly, a 3D model was simulated using IMP,^[^ ^73^ ^]^ and the reference and scHi‐C data were simulated as follows.^[^ ^74^ ^]^ For reference Hi‐C, reads were sampled from any two genome loci *i* and *j* with the weight defined as Weight(*i*, *j*) = 1/distance(*i*, *j*). For scHi‐C data, reads from any two genome loci *i* and *j* were sampled with the weight defined as Weight*(i*, *j*) = *D*‐distance(*i, j*), where *D* is a threshold. Only genome loci having Euclidean distance less than *D* were considered to be contacting. The simulation generated data with 40 kb resolution, and the Hi‐C contact matrix was used for actual TLD detection (see details of simulation from the original paper^[^ ^42^ ^]^).

### Execution of TLD Predictors

All test TLD predictors were run with default settings, except for Higashi,^[^ ^43^ ^]^ which is set to be nbr = 4 for multicell datasets and nbr = 0 for downsampled and simulated Hi‐C data. embedding_epoch = 1 and no_nbr_epoch = 20 were also set for downsampled and Hi‐C data were simulated to save time, and, as suggested by the authors, the TADs of bulk Hi‐C data were called by the function call_tads in Higashi codes. CPU times can be found in Table S5 of the Supporting Information.

### Adjusted Mutual Information (*T*, *K*)

Mutual information MI (*T*, *K*) was defined as

(8) $$\mathrm{MI}\left(T,K\right)=\sum_{i=1}^{n}\sum_{j=1}^{m}P\left(i,j\right)\log\left(\frac{P\left(i,j\right)}{P\left(i\right)P^{\prime }\left(j\right)}\right)$$

where

(9) $$P\left(i\right)=\left|T_{i}\right|/N;P^{\prime }\left(j\right)=\left|K_{j}\right|/N;\;P\left(i,j\right)=T_{i}\cap K_{j}/N$$

Then, the adjusted mutual information AMI (*T*, *K*) was defined as

(10) $$\mathrm{AMI}\left(T,K\right)=\frac{\mathrm{MI}\left(T,K\right)-E\left\{MI\left(T,K\right)\right\}}{\max\left\{H\left(T\right),H\left(K\right)\right\}-E\left\{\mathrm{MI}\left(T,K\right)\right\}}$$

where *H* denotes the Shannon entropy, and *E* denotes expectation. AMI was calculated by the function adjusted_mutual_info_score in the Python module sklearn.metrics. In real calculation, all predicted TLDs and intermediate windows of TLDs are included in *T* and *K*.

### Weighted Similarity WS (*T*, *K*)^[^ ^33^ ^]^

The weighted similarity WS (*T*, *K*) was defined as

(11) $$\mathrm{WS}\left(T,K\right)=\frac{\sum_{j=1}^{m}S_{K}^{T}\left(j\right)*\left|K_{j}\right|}{\sum_{j=1}^{m}\left|K_{j}\right|}$$

where

(12) $$S_{K}^{T}\left(j\right)=\max_{i=1}^{n}\;\left\{\frac{\left|T_{i}\cap K_{j}\right|}{\sqrt{\left|T_{i}\right|*\left|K_{j}\right|}}\right\}$$

Because WS is an asymmetric index for similarity, the predicted TLDs from raw data were always put in *T* and the TLDs from downsampled data in *K*, while the intermediate windows of the domains were not included in either *T* or *K*.

### The Enrichment of Structural Proteins and Histone Marks around TLD Boundaries

ChIP‐seq peaks data of structural proteins and histone marks for Nagano's dataset were downloaded from Yue et al.^[^ ^75^ ^]^ ChIP‐seq peaks data for all other datasets were downloaded from ENCODE (www.encodeproject.org).^[^ ^76^ ^]^ To quantify the enrichment of ChIP‐seq peaks around TLD boundaries, the fold change between peaks found at TLD boundaries and those at adjacent flanking regions was calculated.^[^ ^77^ ^]^ The boundary regions were defined as TLD boundaries with two flanking bins, and flanking regions 100 kb and 500 kb away from the TLD boundaries were defined as background. The fold change of peaks is calculated as $\frac{\mathrm{number}\;\mathrm{of}\;\mathrm{peaks}\;\mathrm{per}\;\mathrm{kb}\;\mathrm{in}\;\mathrm{boundary}\;\mathrm{region}}{\mathrm{number}\;\mathrm{of}\;\mathrm{peaks}\;\mathrm{per}\;\mathrm{kb}\;\mathrm{in}\;\mathrm{background}\;\mathrm{regions}}$.

### The Definition of TLD Boundaries

For predictions by score‐based detectors, e.g., IS, deTOKI, scHiCluster, and Higashi, boundaries were defined as the predicted bins, and the fold changes of ChIP‐seq were calculated at the midpoint of the bin. For predictions by network‐based TLD detectors, e.g., deDoc, deDoc2, SpectralTAD, and GRiNCH, boundaries were defined as the edges between two predicted bins, and the fold changes were calculated at the edges.

### Structural Entropy Index

Hi‐C contact matrix *M* is interpreted as a graph and used TLDs predicted by different algorithms to build an encoding tree *T* with height two. The structural entropy of *M* given the encoding tree *T* (2D structural entropy) was then calculated. The vertices in the gap regions were put directly on the root *λ* of *T* since these vertices do not belong to any communities.

### Modularity Index

For a Hi‐C contact matrix *M*, the diagonal elements with zero were replaced, and the remaining elements were log2 processed. Modularity was defined as

(13) $$Q=\frac{1}{2m}\sum_{ij}\left(M_{ij}-\frac{k_{i}k_{j}}{2m}\right)\delta_{\sigma_{i}\sigma_{j}}$$

where *i* and *j* are bin indices of *M*, *k_i_* and *k_j_* are the degrees of the vertices, and $m=\frac{1}{2}\sum_{i}k_{i}$ is the total number of edges in the network.^[^ ^78^ ^]^ The value of Kronecker delta $\delta_{\sigma_{i}\sigma_{j}}$ equals 1 if *σ*_*i*_ = *σ*_*j*_, where the label *σ*_*i*_ denotes the community label of bin *i*. For the gap region recognized by algorithms, each single locus of the vertices in these regions is taken as a community.

### Embedding and Clustering of Single Cells

The visualization of embedded single cells of TLDs predicted by deDoc2 utilized the result of deDoc2 with RWR imputation, i.e., 4238 cells and 1171 cells used in Lee's and Nagano's datasets, respectively. The AMI and CROC to quantify the embedding used 400 and 560 randomly selected cells in Lee's and Nagano's datasets, respectively.

### Visualization of Embedded Single Cells

Following the visualization method, as described in Zhou et al.,^[^ ^41^ ^]^ 2D embedding was performed using TLD predictions as text‐document input on Lee's dataset. For Nagano's dataset, one step was omitted in the above visualization method, i.e., harmonypy,^[^ ^79^ ^]^ as notable batch effect was spotted which may negatively affect the dimension‐reducing results. Last, t‐distributed stochastic neighbor embedding (T‐SNE) was applied to the dimensionality‐reduced matrix to get a 2D map of the data for visualization by the Python module MulticoreT‐SNE.

### CROC

The visualization result described above was used to calculate CROC^[^ ^57^ ^]^ to evaluate the quality of embedding from TLD boundaries of a cell‐cycle dataset. For a multiclass dataset, CROC was calculated for one class at a time by taking all remaining classes as negative points. For a circular 2D cell embedding, a unique angle *θ* is obtained for each cell. It is assumed that the angles of a class follow a von Mises distribution and calculated the mean angle θ* as the true positive label of the class; then the difference between θ* and every angle *θ*_*i*_ of a data point was taken as the predicted score of this data point. The ROC calculation was then performed by sklearn.metrics.roc_auc_score. To evaluate the embedding of all cell types, the average CROC was calculated across these types.

### Evaluation of the Embedding of Noncircular Datasets

To evaluate the embedding of noncircular datasets, the embedding was clustered and the AMI score was calculated,^[^ ^52^ ^]^ After the first dimensionality reduction as it was in the visualization step, *k*‐means clustering was applied on the first 15 dimensions of the dimensionality‐reduced matrix and the AMI score was calculated by the Python module sklearn.metrics.adjusted_mutual_info_score.

### Shuffled Control of TLD WS Score

To assess the significance of WS score of TLDs, the locations of TLD boundaries were randomized preserving the distributions of TLD sizes and the number of TLDs per chromosome. Each randomization was performed 2500 times on randomly chosen cells.

### Hierarchical TLD Assessment

The WS metric was utilized to assess the similarity of hierarchical TLDs between two groups. Specifically, the WS of nested TLDs was first calculated within each group. Next, similar nested TLD pairs were identified between groups *T* and *K*, where *T*1 and *K*2 obtained the maximum *S* value. Subsequently, the WS values of subTLDs within all similar nested TLD pairs were computed and the average value was obtained, which represents the WS of subTLDs within nested TLDs of groups *T* and *K*.

Each nested TLD is regarded as an independent matrix and modularity is calculated according to the division of the subTLDs. TLD modularity is the average modularity of all nested TLDs in the cell.

TLD‐adjR^2^ was adopted from An et al,^[^ ^34^ ^]^ and it can measure the proportion of Hi‐C signal variation explained by TAD calls. For any given genomic region and given genome distance, the TLD‐adjR^2^ is defined as

(14) $$\mathrm{TLD}-\mathrm{adj}R^{2}=1-\frac{\frac{1}{n-p-1}\sum_{i=1}^{n}{\left(Y_{i}-{\hat{Y}}_{i}\right)}^{2}}{\frac{1}{n-1}\sum_{i=1}^{n}{\left(Y_{i}-\bar{Y}\right)}^{2}}$$

where *Y_i_* denotes the contact number of the *i*th bin, *n* denotes the number of bins at the same genomic distance as this bin, and *p* denotes the number of subTLDs larger than, or equal to, the genomic distance. For bins within subTLD, ${\hat{Y}}_{i}$ denotes the average contact frequency at a given genomic distance within that subTLD. For those bins not in any subTLDs, ${\hat{Y}}_{i}$ is the average of contact frequency in the gap region at that genomic distance, and $\bar{Y}$ denotes the overall mean contact frequency across all bins at a given genomic distance.

### Identification of TLD Cliques

The pipeline for TLD clique identification was adopted from Paulsen et al,^[^ ^59^ ^]^ the TAD was only modified with TLD during the whole process, and the cliques were only analyzed with more than 4 TLDs.

### The Enrichment of Inter‐TLD Contacts in TLD‐Cliques

Three rules from Paulsen et al. were adopted:^59^ i) the permuted clique was on the same chromosome as the observed clique; ii) sizes of TLDs in a clique were kept; iii) genomic distance between consecutive TLDs in the clique was maintained. The start position of a TLD clique was randomly positioned on the same chromosome and TLD size and inter‐TLD distances were shuffled to construct a permuted TLD clique. In total, 250 permutations were carried out and the expected inter‐TLD contacts are the mean inter‐TLD contacts of the 250 permuted cliques. The enrichment of inter‐TLD contacts in TLD‐cliques is calculated as (observed inter‐TLD contacts)/(expected inter‐TLD contacts).

### Cell Type‐Specific Highly Expressed Genes and Nested TLD Boundaries

To get cell type‐specific highly expressed genes, the single‐cell expression data of Astro, MG, L2/3, and Vip were first obtained from the single‐cell RNA‐seq dataset of brain (https://portal.brain‐map.org/atlases‐and‐data/rnaseq/human‐multiple‐cortical‐areas‐smart‐seq). Then, if the expression level of gene A in certain cell type was higher than that of the other three cell types, gene A was regarded as the corresponding high‐expression gene of this cell type.

As for cell type‐specific nested TLD boundaries, the frequency of occurrence of a boundary in each of the four cell types was first calculated. If the frequency of occurrence of a boundary was higher than the mean plus three standard deviations, it was called a high frequency boundary. If the three bins before and after this position did not have a high frequency boundary in other cell types, then that boundary was set as the boundary specific to this cell type.

## Conflict of Interest

The authors declare no conflict of interest.

## Author Contributions

A.L., G.Z., and H.W. contributed equally to this work. A.L., G.Z., and Z.Z. conceived this project. G.Z. and H.W. performed the experiments. G.Z., H.W., and X.L. analyzed data. A.L., G.Z., H.W., and Z.Z. prepared the manuscript. All authors read and approved the final manuscript.

## Supporting information

Supporting Information

Supporting Information

Supporting Information

Supporting Information

Supporting Information

Supporting Information

Supporting Information

## Data Availability

The data that supports the findings of this study are available in the Supporting Information of this article.
